# NUP98‐HOXA10hd fusion protein sustains multi‐lineage haematopoiesis of lineage‐committed progenitors in transplant setting

**DOI:** 10.1111/cpr.12885

**Published:** 2020-07-29

**Authors:** Yong Dong, Kaitao Wang, Qitong Weng, Tongjie Wang, Peiqing Zhou, Xiaofei Liu, Yang Geng, Lijuan Liu, Hongling Wu, Jinyong Wang, Juan Du

**Affiliations:** ^1^ CAS Key Laboratory of Regenerative Biology Joint School of Life Sciences Guangzhou Institutes of Biomedicine and Health, Chinese Academy of Sciences Guangzhou Medical University Guangzhou China; ^2^ Guangdong Provincial Key Laboratory of Stem Cell and Regenerative Medicine Guangzhou Institutes of Biomedicine and Health Guangzhou China; ^3^ University of Chinese Academy of Sciences Beijing China; ^4^ Institute of Blood Transfusion Chinese Academy of Medical Sciences & Peking Union Medical College (CAMS & PUMC) Chengdu China; ^5^ Guangzhou Regenerative Medicine and Health Guangdong Laboratory Guangzhou China; ^6^ Institute for Stem Cell and Regeneration Chinese Academy of Sciences Beijing China

## Abstract

**Objectives:**

Exploring approaches of extending the haematopoiesis time window of MPPs and lineage‐committed progenitors might produce promising therapeutic effects. NUP98‐HOXA10hd (NA) fusion protein can expand long‐term haematopoietic stem cells (HSCs) and promote engraftment competitiveness without causing obvious oncogenesis. Our objectives were to investigate the roles of NA fusion protein in MPP and downstream lineage‐committed progenitor context.

**Material and Methods:**

300 sorted MPPs (Lin^−^CD48^−^c‐kit^+^Sca1^+^CD135^+^CD150^−^) were mixed with 5 × 10^5^ total BM helper/competitor cells and injected into irradiated recipients. For secondary transplantation, 5 × 10^6^ total BM cells from primary recipient mice were injected into lethally irradiated recipients. NA‐MPP recipient mice were sacrified for flow cytometric analysis of bone marrow progenitors at indicated time points. Sorted MPPs and myeloid progenitors were used for RNA‐seq library preparation.

**Results:**

We showed that NA‐expressing MPPs achieved significantly longer multi‐lineage haematopoiesis (>44‐week) than natural MPPs (20‐week). NA upregulated essential genes regulating long‐term haematopoiesis, cell cycle, epigenetic regulation and responses to stress in MPPs. These molecular traits are associated with the earlier appearance of a Sca1^‐^c‐kit^+^ myeloid progenitor population, and more abundant cellularity of lineage‐committed progenitor as well as bone marrow nucleated cells. Further, the NA‐derived primary bone marrow cells, which lack NA‐LSK cells, successfully repopulated secondary multi‐lineage haematopoiesis over 20 weeks.

**Conclusions:**

This study unveiled that NA fusion protein promotes MPP and lineage‐committed progenitor engraftment via extending long‐term multi‐lineage haematopoiesis.

## Graphical abstract

Yong Dong et al. show that MPPs overexpressing NUP98‐HOXA10hd fusion protein (NA) generate abundant lineage committed progenitors and sustain long‐term multi‐lineage hematopoiesis in transplant setting. Overexpression of NA in MPPs offer promising means to involve MPPs in clinical transplant settings.

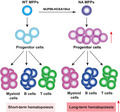

## 1 INTRODUCTION

Allogeneic stem cell transplantation is widely used for blood disorders, such as leukaemia, myeloproliferative diseases, anaemia. However, lack of timely available HLA matched‐donor hindered the application of allogeneic transplantation in clinical applications. Cord blood banks hold the potential to serve as the cell source for transplantation, while the insufficient haematopoietic stem cell number in one unit of cold blood become the main obstacle for the application of cord blood. One solution is to expand haematopoietic stem cell in vitro prior to transplantation. Great efforts have been made to achieve haematopoietic stem cell (HSC) expansion, including the use of small molecules, such as SR1, UM171, UM729, as well as the introduction of intrinsic regulators into HSC‐enriched stem cell population, HOXB4 for example.^1^, ^2^, ^3^, ^4^, ^5^, ^6^ However, the clinical efficacy of these approaches remains unknown and need further investigations. Another possible solution is to enhance self‐renewal potential of progenitors or generate abundant progenitor cells to reconstitute haematopoiesis. If this progenitor population could successfully sustain multi‐lineage haematopoiesis for a prolonged time period, infusion of enhanced progenitor cells regularly could be an alternative option when allogeneic donor is unavailable. Until now, limited but encouraging success has been achieved in this aspect. Ectopic expression of transcription factor Sox17 via retroviruses has been demonstrated to increase the self‐renewal potential of multipotent progenitors (MPPs) and therefore conferred on MPPs the potential for long‐term multi‐lineage reconstitution.^7^ Another study has successfully conferred long‐term repopulating ability on MPPs with a single miRNA, miR‐125a. The enforced expression of miR‐125a endowed MPPs with enhanced self‐renewal potential, resulting in robust long‐term multi‐lineage repopulation.^8^

NUP98‐HOXA10hd fusion gene (NA) has been shown to be potent for haematopoietic stem cells to expand, survive under stress^9^, ^10^ and promote engraftment competitiveness.^11^ However, whether the ectopic expression of NA in MPPs and lineage‐committed progenitors could confer long‐term multi‐lineage haematopoiesis remains unknown. In this study, we explored the roles of NA fusion protein in MPPs and their downstream lineage‐committed progenitor context. We used the MPPs sorted from NA compound mice for transplantation assays, which enable the stable overexpression of Nup98‐Hoxa10 fusion gene from Rosa26 locus instead of retroviral transduction to avoid risk of insertional mutagenesis. Here, we found that ectopic expression of NA fusion protein in MPPs conferred long‐term multi‐lineage haematopoiesis in recipient mice, offering promising means to involve MPPs to augment cell source in clinical transplantation settings.

## 2 MATERIAL AND METHODS

### 2.1 Mice

NA^LSL/+^ mice were generated by targeting a mouse ES line (C57BL/6 line, CD45.2^+^, Beijing Biocytogen Co., Ltd.) through homologous recombination as previously described.^11^ C57BL/6 (CD45.2, CD45.1) and Vav‐Cre strain (CD45.2) mice were purchased from the Jackson Laboratory. NA^LSL/+^ mice were subsequently bred with Vav‐Cre mice to generate NA^LSL/+^; Vav‐Cre (NA) mice. Mice were housed in the SPF grade animal facility of the Guangzhou Institution of Biomedicine and Health, Chinese Academy of Science (GIBH, CAS, China). All animal experiments were approved by the Institutional Animal Care and Use Committee of Guangzhou Institutes of Biomedicine and Health (IACUC‐GIBH).

### 2.2 Transplantation

Adult C57BL/6 recipient mice (CD45.2^+^, 8‐10 weeks old) were irradiated with 2 doses of 4.75 Gy (RS 2000, Rad Source) for a 4‐hour interval. For MPP transplantation assay, 300 sorted MPP cells (Lin^−^CD48^−^c‐kit^+^Sca1^+^CD135^+^CD150^−^) were mixed with 5 × 10^5^ total BM helper/competitor cells or Sca1^−^ BM helper cells and subsequently injected into the retro‐orbital vein of the irradiated recipients. The transplanted mice were maintained on trimethoprim‐sulphamethoxazole‐treated water for 2 weeks. After transplantation, peripheral blood was obtained from the retro‐orbital vein regularly for flow cytometric analysis. For secondary transplantation, 5 × 10^6^ total BM cells from primary recipient mice were injected into the retro‐orbital vein of the lethally irradiated recipients.

### 2.3 Flow cytometry analysis

Antibodies for haematopoietic lineage and haematopoietic progenitors or stem cells analysis: CD45.2(104), CD2 (RM2‐5), CD3e (145‐2C11), CD4 (RM4‐5), CD8a (53‐6.7), Ter119 (TER‐119), Mac1 (M1/70), B220 (6B2), Gr1 (RB6‐8C5), CD19 (1D3/CD19), CD90.2 (53‐2.1), CD48 (HM48‐1), IL‐7R (A7R34), Sca1 (E13‐161.7), c‐Kit (2B8), CD34 (RAM34) and CD16/32 (93) antibodies were purchased from eBiosciences, and CD150 (TC15‐12F12.2) was purchased from Biolegend. DAPI (#D9542‐50MG) was purchased form sigma.

All the nucleated cells harvested from mouse tissues were first treated with ACK solution, and then blocked with CD16/32 antibody except for myeloid progenitor staining. For lineage staining, blocked nucleated cells were incubated with antibody mixture of CD19‐FITC, Mac1‐PE‐Cy7 and CD90.2‐APC. For myeloid progenitors (MP) staining, BM cells were stained with antibody mixture of Lin (CD2, CD3e, CD4, CD8a, Ter119, Mac1, B220, Gr1)‐FITC, Sca1‐PerCP‐Cy5.5, c‐Kit‐APC‐eFluor®780, CD34‐Alex700 and CD16/32‐PE‐Cy7. For lymphocyte progenitors staining, BM cells were stained with the antibody mixture of Lin (CD2, CD3e, CD4, CD8a, Ter119, Mac1, B220 and Gr1)‐FITC, IL‐7R‐APC, Sca1‐PerCP‐Cy5.5, c‐Kit‐APC‐eFluor^®^ 780. For MPP staining, BM cells were stained with the antibody mixture of Lin (CD2, CD3e, CD4, CD8a, Ter119, Mac1, B220 and Gr1)‐FITC, CD48‐FITC, Sca1‐PerCP‐Cyanine5.5, c‐Kit‐APC‐eFluor^®^ 780, CD45.1‐PE (only in CD45.1^+^ WT MPP recipients) and CD150‐PE‐Cy7. For LSK staining, BM cells were stained with the antibody mixture of Lin (CD2, CD3e, CD4, CD8a, Ter119, Mac1, B220 and Gr1)‐FITC, Sca1‐PerCP‐Cyanine5.5, c‐Kit‐APC‐eFluor^®^780, CD45.1‐PE (only in CD45.1^+^ WT MPP recipients). Finally, all the cells were resuspended in DAPI solution.

The stained cells were analysed on LSR Fortessa (BD Bioscience), and the data were analysed using Flowjo software (FlowJo).

### 2.4 RNA‐Seq and data analysis

MPPs were sorted separately into 200 µL DPBS‐BSA buffer (0.5% BSA) using 1.5 mL EP tube. Sequencing libraries of MPPs were generated as previously described for HSC.^11^ Briefly, cDNA of sorted 1000 MPPs aliquots was generated, amplified^12^ and used for sequencing library preparation with illumina Nextera XT DNA Sample Preparation Kit (FC‐131‐1096). For MP sequencing, 1 × 10^5^ myeloid progenitor cells (Lin^−^c‐kit^+^Sca1^−^) were sorted as one sample from bone marrow nucleated cells of respective recipient mice. Donor‐derived NA MP cells (Tdtomato^+^) were sorted from NA MPP recipient mice 4 months post‐transplantation, while WT MP cells (CD45.2^+^) were obtained from control group received WT MPP transplantation at the same time point. Following RNA extraction with RNeasy micro kit (QIAGEN), total RNA of each sorted myeloid progenitor sample was used for sequencing library preparation with illumina Truseq RNA Sample Preparation Kit (RS‐122‐2001). All libraries were sequenced by illumina sequencer NextSeq 500 (illumina). The fastq raw data files were generated using illumina bcl2fastq software and uploaded to Gene Expression Omnibus public database (GSE146781). Alignment, normalization of genes expression and differential expression genes (DEGs) analysis were performed by RSEM and DESeq2. GO enrichment analysis was performed with clusterProfiler package. Gene set enrichment analysis (GSEA) was performed as described.^13^ Heatmaps were plotted using gplots (heatmap.2) and ggplot2 package.

### 2.5 Statistical analysis

Statistical analysis was performed with SPSS (SPSS v.23, IBM Corp.). Normal distribution of data was tested with SPSS applying Shapiro‐Wilk normality test. The data were represented as mean ± SEM. Two‐tailed independent Student's *t* tests were performed for comparison of two groups of data. *P* values <.05 were considered statistically significant (*P < .05, **P < .01 and ***P < .001).

## 3 RESULTS

### 3.1 NA‐overexpressing MPPs sustain long‐term multi‐lineage haematopoiesis in primary mice

**Figure 1:**
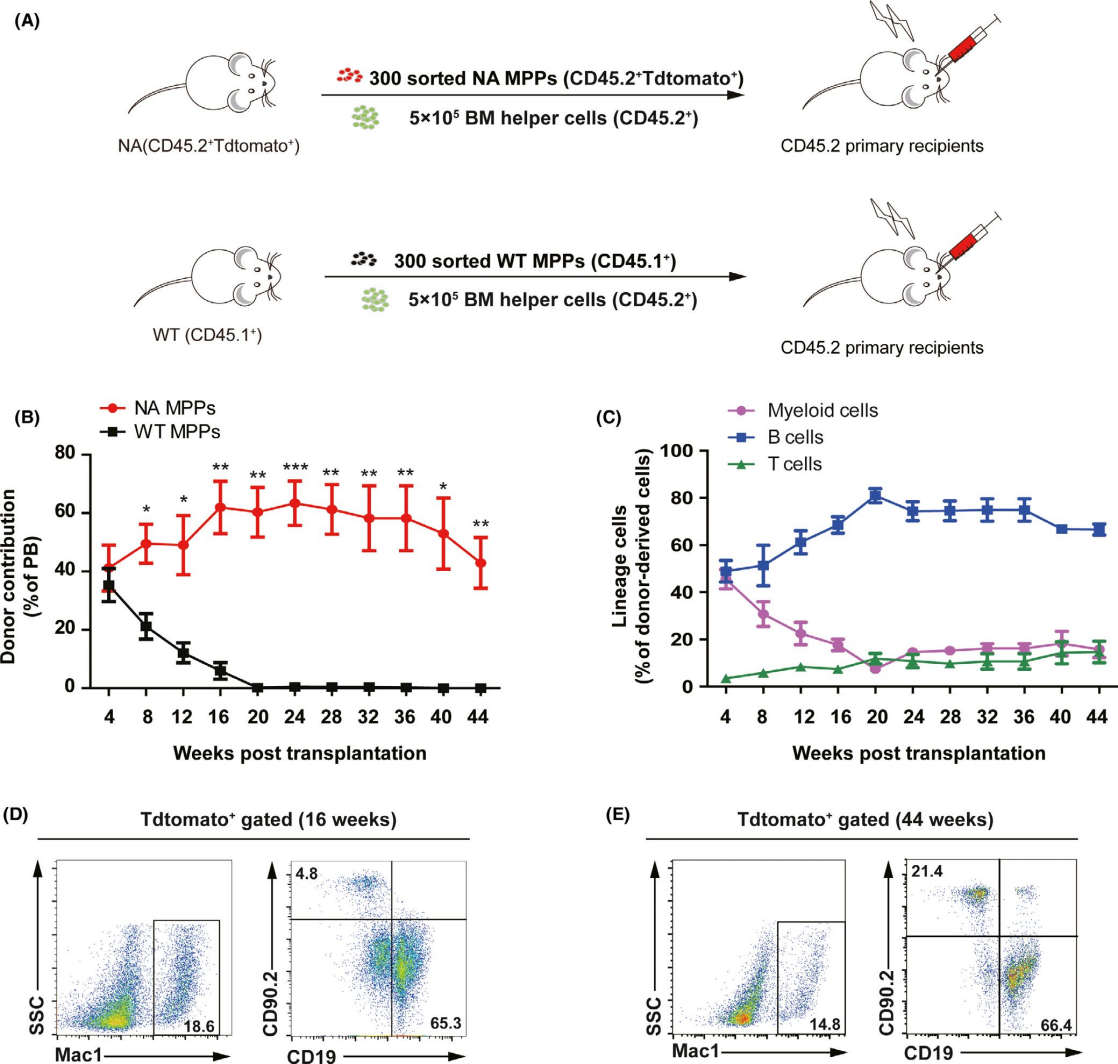
MPPs overexpressing NA support long‐term multi‐lineage haematopoiesis in primary recipient mice. A, Schematic strategy of MPP transplantation. For MPP transplantation assay, 300 sorted MPP cells (Lin^−^CD48^−^c‐kit^+^Sca1^+^CD135^+^CD150^−^) were mixed with 5 × 10^5^ total BM helper/competitor cells and subsequently injected into the retro‐orbital vein of the irradiated recipients. B, Dynamic contribution of donor‐derived white blood cells (Tdtomato^+^ or CD45.1^+^) in peripheral blood of recipient mice at different time points post‐transplantation. C, Dynamic donor‐derived multi‐lineage reconstitution for NA MPPs group in (B). Four mice in each group were bled regularly up to 44 wk post‐transplantation. Lineage analysis of donor‐derived white blood cells in PB at 16 wk (D) and 44 wk (E) post‐transplantation. Flow plots from one representative mouse of NA MPPs group are shown. Data are represented as means ± SEM. Unpaired Student's *t* test (two‐tailed) was performed. n = 4 mice, *P < .05, **P < .01, ***P < .001

We have previously established NA‐overexpression mouse model and found that ectopic expression of NA confers engraftment competitiveness of LT‐HSCs in competitive transplantation assay.^11^ We further investigated the cell‐context roles of NA in MPPs. We directly sorted 300 MPP (Lin^−^CD48^−^c‐kit^+^Sca1^+^CD135^+^CD150^−^) cells (Figure S1) from bone marrow nucleated cells of NA mice, and transplanted them together with 5 × 10^5^ total bone marrow helper/competitor cells into lethally irradiated recipient (Figure 1A). Recipient mice were bled regularly to monitor donor contribution and donor‐derived lineages. Strikingly, 300 NA MPPs continuously contributed to multi‐lineage reconstitution over 44 weeks. Donor contribution started at 41% at 4‐week time point, increased gradually and persisted around 60% from week‐16 to week‐36 and decreased to 40% at 44‐week time point. In contrast, donor contribution of WT MPP recipient mice peaked at 35% at 4‐week time point, dramatically decreased and became undetectable from 20‐week time point (Figure 1B). NA MPPs contributed to myeloid, B and T cell lineages at all time points (Figure 1C‐E). Expectedly, non–NA‐derived multi‐lineage haematopoiesis was observed in recipient mice (Figure S2). Interestingly, when 300 NA MPPs were transplanted with 5 × 10^5^ Sca1^−^ bone marrow helper cells, we also observed donor‐derived long‐term multi‐lineage reconstitution in recipient mice and the donor contribution started from 83% at week‐5 and decreased gradually to 61% at week‐40. Moreover, NA MPPs contributed to all lineages including myeloid, B and T cells (Figure S3). Taken together, our data demonstrated that NA‐overexpressing MPPs repopulated long‐term multi‐lineage haematopoiesis both with and without of WT HSC/MPP competitors in primary mice.

### 3.2 NA upregulates genes regulating long‐term haematopoiesis, cell cycle, epigenetic regulation and responses to stress in MPPs

**Figure 2:**
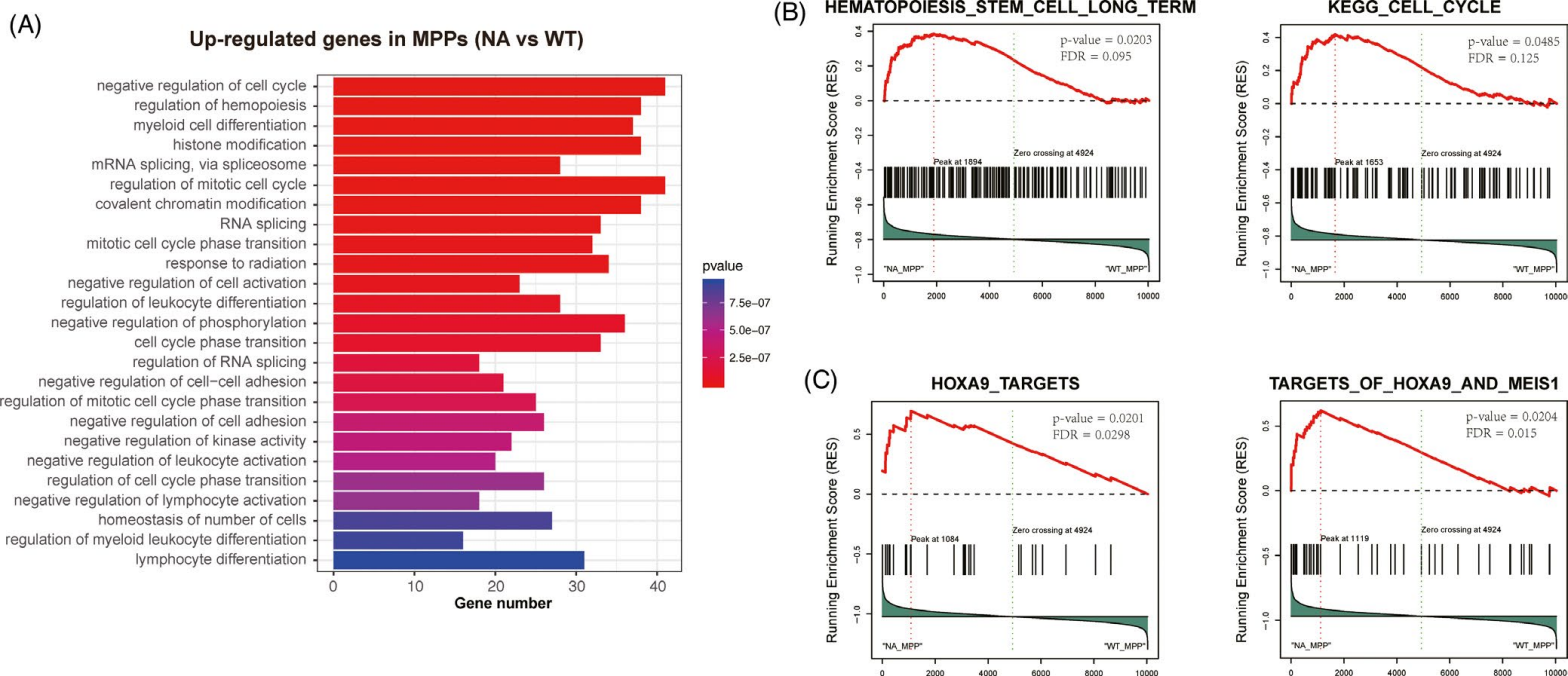
Transcriptome profiling of NA MPPs. A, Gene ontology enrichment analysis of upregulated genes. One thousand sorted MPP were sorted as each sample of sequencing library preparation. Genes with at least 2‐fold upregulation over WT MPPs were included for GO enrichment analysis. B and C, GSEA analysis of gene sets of hematopoiesis_stem_cell_long_term, KEGG_cell_cycle (B), Hoxa9 targets and targets_of_Hoxa9_and_Meis1 (C) in transcriptome of NA MPPs compared with WT MPPs. Gene sets were obtained from the data set of c2.all.v5.2 symbols of the GSEA website (https://gsea‐msigdb.org). Selected pathways with significant changes are shown (FDR < 0.25, *P* < .05)

In order to investigate how NA altered MPPs at molecular level, we sorted MPPs from NA and WT mice for transcriptome analysis. Since one thousand cell input for RNA‐Seq produced reads uniformly covers all transcripts,^14^ we sorted one thousand MPP aliquots for RNA‐Seq analysis. Differential gene expression analysis identified 2347 differential expression genes (DEGs) in NA MPPs compared with WT MPPs (Table S1). GO enrichment analysis of upregulated DEGs (>2 fold) using the NA MPPs showed that these DEGs were largely involved in the regulation of cell cycle, epigenetic regulation, responses to stress, RNA splicing and regulation of signalling pathways (Figure 2A). Further gene set enrichment analysis (GSEA) showed NA regulated genes were enriched for gene sets of long‐term haematopoiesis and cell cycle (Figure 2B). Overexpression of Hoxa9 has been reported to expand HSC compartment and results in lympho‐myeloid long‐term repopulation^15^; meanwhile, Hoxa9 deficiency impairs the multi‐lineage repopulating ability of HSC.^16^ Interestingly, NA regulated genes were enriched for Hoxa9_targets and targets_of_Hoxa9_and_Meis1 (Figure 2C). Collectively, our data imply that NA endows MPPs with the capability to support long‐term haematopoiesis through regulating gene involving in the regulation of long‐term haematopoiesis, cell cycle regulation, epigenetic regulation and response to stress.

### 3.3 NA‐MPPs efficiently generated abundant lineage‐committed progenitors

**Figure 3:**
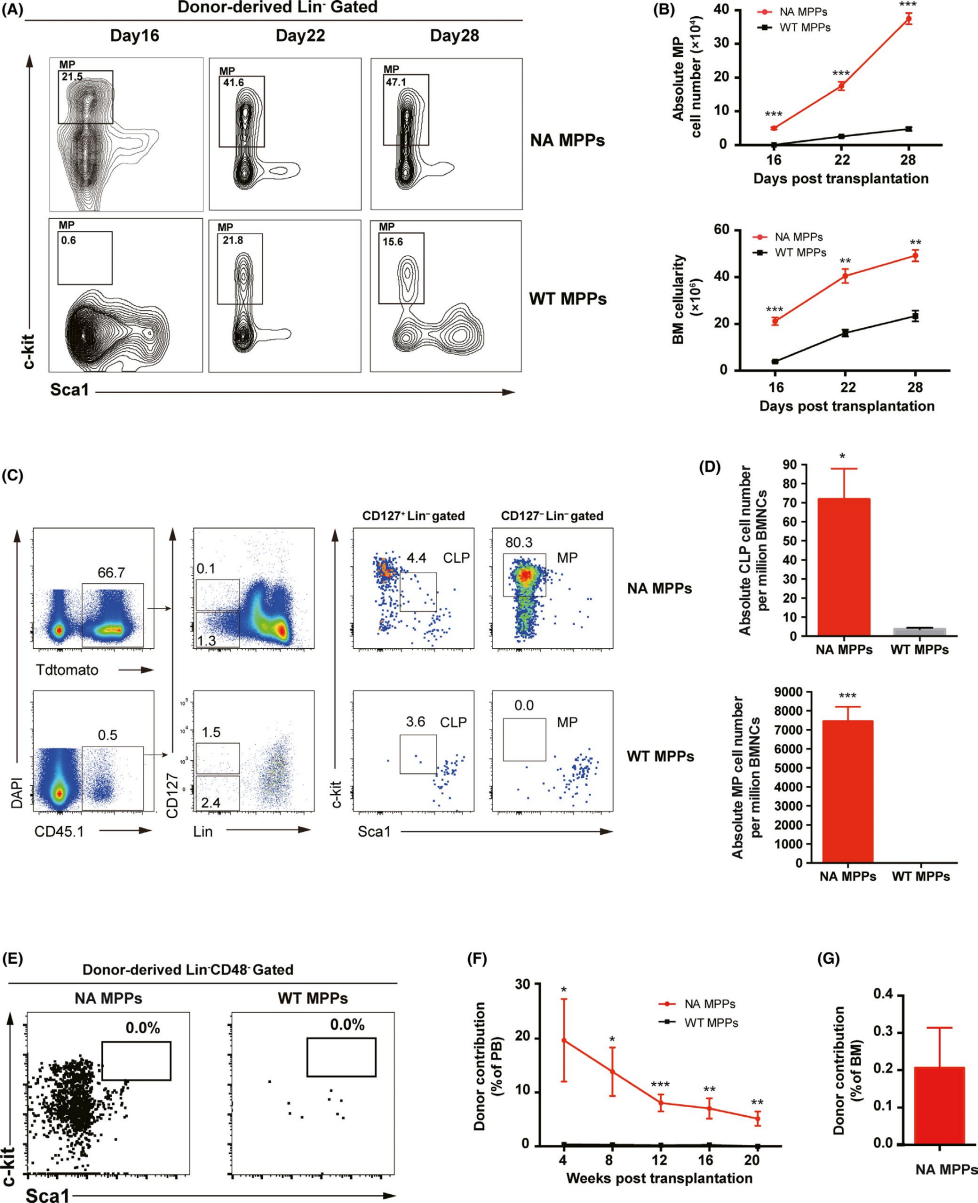
NA MPPs give rise to abundant lineage‐committed progenitors in primary recipient mice. NA‐MPP recipient mice were sacrified for bone marrow progenitor analysis 20 wk post‐transplantation if not otherwise indicated. A, Flow cytometric analysis of donor‐derived myeloid progenitor cells in primary recipient mice. Recipient mice were sacrified for analysis at indicated time points post‐transplantation. B, Absolute number of MP cells (upper) and bone marrow cellularity (lower) of donor‐derived white blood cells. Donor‐contributed percentage were analysed by flow cytometry and used for calculation. (n = 3) (C) Flow cytometric analysis of donor‐derived CLP and MP cells in primary recipient mice. Flow plots from one representative mouse of each group are shown. D, Absolute number of donor‐derived CLP and MP cells per million BMNCs. E, Flow cytometric analysis of donor‐derived LSK cells in primary recipient mice. Flow plots from one representative mouse of each group are shown. F, Dynamic contribution of donor‐derived white blood cells (Tdtomato^+^ or CD45.1^+^) in peripheral blood of secondary recipient mice at different time points post‐transplantation. 5 × 10^6^ total BM cells from primary recipient mice were injected into the retro‐orbital vein of the lethally irradiated recipients for secondary transplantation (n = 4). G, Donor contribution in bone marrow of secondary recipient mice. Secondary recipient mice of NA MPPs group were sacrificed for analysis 20 wk post‐transplantation (n = 3). Data are represented as means ± SEM. Unpaired Student's *t* test (two‐tailed) was performed. *P < .05, **P < .01, ***P < .001. BMNCs, bone marrow nucleated cells; CLP, common lymphoid progenitors; MP, myeloid progenitors

To investigate whether NA‐MPPs produce abundant lineage‐committed progenitors to sustain long‐term haematopoiesis, we investigated the bone marrow progenitor components in primary MPP recipient mice. Firstly, we transplanted 300 sorted MPPs and analysed the bone marrow myeloid progenitor compartment at day 16, 22, 28 post‐transplantation. Not only we observed donor‐derived MPs at an earlier time point (day‐16), but also at higher ratios compared with control (Figure 3A). As expected, NA MPPs produced more myeloid progenitor cells and eventually output much more mature white blood cells which contributed to higher number of bone marrow cellularity at day 16, 22, 28 after MPP transplantation (Figure 3B). Since NA MPPs achieved long‐term multi‐lineage haematopoiesis, we further analysed lineage‐committed progenitor population including common lymphoid progenitors and myeloid progenitors at week‐20. Flow cytometric analysis of bone donor‐derived progenitors showed that both percentage and absolute number of common lymphoid progenitors and myeloid progenitors were significantly higher compared with control (Figure 3C,D). To investigate the possibility that NA might induce dedifferentiation of MPP back to LT‐HSCs, we analysed the LSK compartment in recipient mice at week‐20. Interestingly, no detectable donor‐derived LSK population persisted at week‐20 (Figure 3E). To further test whether NA‐MPPs possess long‐term capability of haematopoiesis, we transplanted 5 million total BM cells from primary NA‐MPP recipient mice into lethally irradiated secondary recipient mice and bled these mice to monitor NA‐MPP‐derived multi‐lineage haematopoiesis. Interestingly, donor contribution in PB of secondary recipients was 19% at 4 weeks post‐transplantation, decreased gradually to 6% at week‐20 (Figure 3F). We sacrificed these secondary recipient mice at week‐20 to examine the NA MPP‐derived donor contribution. Flow cytometric analysis of bone marrow nucleated cells of these secondary recipients revealed less than 0.4% donor contribution (Figure 3G). Taken together, our results suggest that NA MPPs achieve long‐term haematopoiesis via generating abundant lineage‐committed progenitors rather than acquiring self‐renewal capability.

### 3.4 NA MPs upregulate genes involving in regulation of phosphorylation and myeloid differentiation

**Figure 4:**
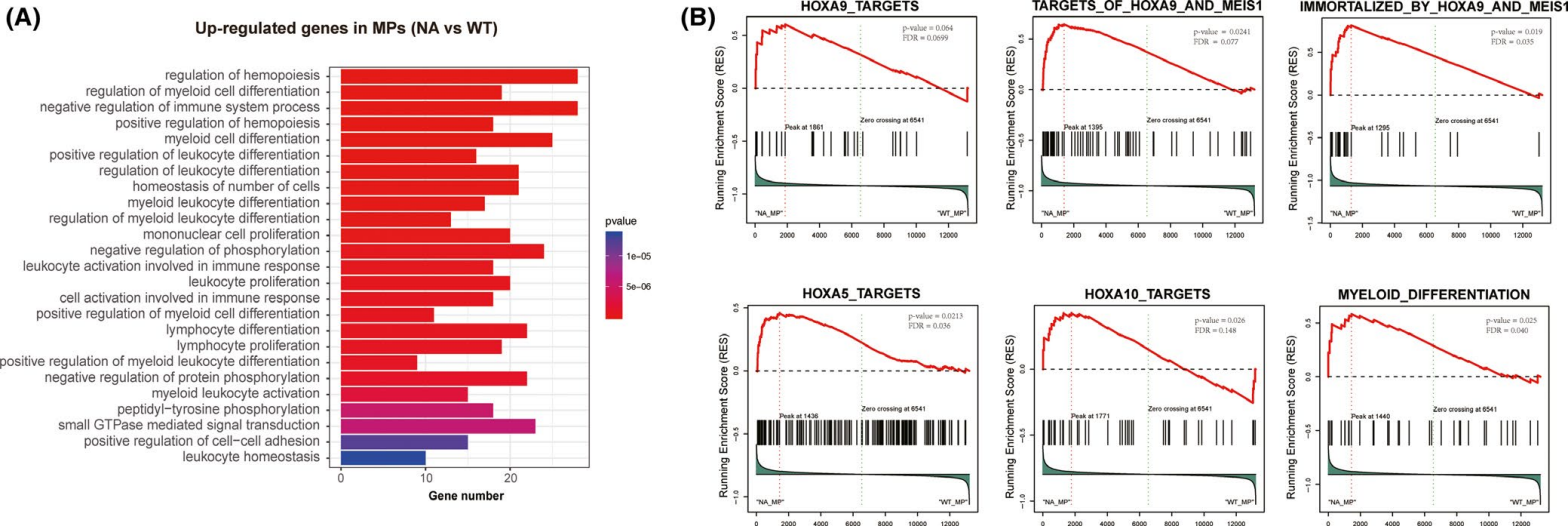
Transcriptome profiling of NA MPs. A, Gene ontology enrichment analysis of upregulated genes in NA MPs. MPs were sorted from primary recipient mice to construct RNA‐seq libraries. Genes with at least 2‐fold change over WT MPs were included for GO enrichment analysis. B, GSEA analysis of gene sets of Hoxa9_targets, targets_of_Hoxa9_and_meis1, immortalized_by_Hoxa9_and_Meis1, Hoxa5_targets, Hoxa10_targets and myeloid_differentiation in transcriptome of NA MPs compared with WT MPs. Gene sets were obtained from the data set of c2.all.v5.2 symbols of the GSEA website (https://gsea‐msigdb.org). Selected pathways with significant changes are shown (FDR < 0.25, *P* < .05)

Donor‐derived progenitor cells supported haematopoiesis up to 44 weeks while there was no stem cell‐enriched LSK population, indicating that these progenitor cells gained new function features. To gain molecular insights of NA‐expressing lineage‐committed progenitor cells, we investigated the transcriptome patterns of myeloid progenitor cells by RNA‐seq analysis. Differential gene expression analysis identified 3051 differential expression genes (DEGs) in NA MPs compared with WT control (Table S2). GO enrichment analysis of upregulated DEGs (>2 fold) using the NA MPs showed that these DEGs were largely involved in the regulation of haematopoiesis, homoeostasis, phosphorylation in addition to myeloid cell differentiation (Figure 4A). Notably, further gene set enrichment analysis (GSEA) for MPs also identified enrichment of gene sets of Hoxa9_targets as well as other Hox family targets (Figure 4B). Together, these data suggest NA MPs sustain haematopoiesis through actively upregulating genes involving in haematopoiesis, homoeostasis, phosphorylation and myeloid cell differentiation‐related pathways.

## 4 DISCUSSION

In this study, we demonstrated that NA‐overexpressing MPPs can support long‐term multi‐lineage haematopoiesis in primary mice. Few studies have previously reported the robust long‐term multi‐lineage reconstitution induced by enhancing self‐renewal potential of MPPs in recipient mice. Edyta and colleagues achieved increased self‐renewal of MPPs by retroviral ectopic expression of miR125a. However, development of myeloproliferative disease/leukaemia in secondary and tertiary recipients was observed.^8^ Another study successfully employed overexpression of transcription factor Sox17 to confer increased self‐renewal of multipotent progenitor cells to support long‐term multi‐lineage reconstitution. Unfortunately, long‐term ectopic expression of Sox17 eventually led to leukemogenesis.^7^ In our case, long‐term multi‐lineage haematopoiesis is unlikely to be induced by enhanced self‐renewal potential of MPP, as shown by the absence of LSK population in primary recipient mice and the loss of donor contribution in bone marrow of secondary recipient mice. In addition, the NA MPP primary recipient mice as well as the secondary mice remained leukaemia free, demonstrating no obvious oncogenesis of our approach. This is consistent with the previous report that the restriction of NUP98‐fusion to the homeodomain of Hoxa10 blunts leukemogenic potential of Nup98‐Hoxa10.^17^

In our experiment, overexpression of NA in MPPs upregulated genes regulating long‐term haematopoiesis, cell cycle, epigenetic regulation, response to stress and RNA splicing. These molecular signatures could contribute to the capability to sustain long‐term multi‐lineage haematopoiesis of NA MPPs. We have validated the expression of representative genes of leading edge genes for gene sets of cell cycle regulation and targets of Hoxa9 and Meis1 by real‐time PCR (Figure S4). Importantly, further investigation is needed to determine whether changes at transcriptome level ultimately confer alterations of cellular characteristics of NA MPPs, which may contribute to their capacity to sustain long‐term multi‐lineage haematopoiesis. We have examined the cell cycle status of NA MPPs and the results showed NA MPPs was more quiescent compare to control (Figure S5). Further study of other alteration of cellular characteristics of NA MPPs, such as proliferation rate, apoptosis and cell division, would provide more clues on the mechanism how overexpression of NA endows MPPs with the ability to sustain long‐term multi‐lineage haematopoiesis.

We found the complete absence of LSK population at 20 weeks post‐transplantation in primary mice. Yet, the NA MPP recipient mice could sustain haematopoiesis for another 24 weeks as well as support a low but constant donor‐contributed multi‐lineage haematopoiesis in secondary recipients. This could be induced by abundant number of lineage‐committed progenitors generated by NA MPPs. In order to generate enough lineage‐committed progenitors to sustain haematopoiesis, certain number of input NA MPPs are needed. This is supported by our parallel transplantation experiment with 100 NA MPPs and 300 NA MPPs. Transplantation of one hundred NA MPPs was insufficient to maintain steady high level of donor‐contributed haematopoiesis within 20 weeks post‐transplantation while 300 MPPs achieved steady high level of donor‐contributed multi‐lineage reconstitution (Figure S6). In addition, NA may also alter the proliferation and survival of lineage‐committed. Long‐lived progenitor cells have been shown to sustain steady‐state haematopoiesis.^18^ It is possible that NA‐overexpressing lineage‐committed progenitor cells become long‐lived and sustain long‐term multi‐lineage constitution when stem cell containing LSK compartment exhausts. Transplantation of NA MPPs combined with lineage‐tracing strategy will illustrate whether long‐lived committed progenitor cells existed and persisted in primary and secondary transplantation.

Notably, Hoxa9_targets appeared to be common gene sets enriched for GSEA analysis both in NA MPPs and MPs, and it was among the 23 overlapped differentially expressed TFs between MPPs and MPs (Figure S7), indicating it may be the key target of NA across various cellular contexts. Overexpression of Hoxa9 expands HSC compartment and results in lympho‐myeloid long‐term repopulation,^15^ while the loss of Hoxa9 impairs the multi‐lineage repopulating ability of HSC,^16^ implying Hoxa9 could also be an important player as the downstream target of NA in our case. Transplantation assay with NA‐Hoxa9^‐/‐^ MPPs will reveal the dependency of NA on Hoxa9 to exert its role to endow MPPs with the capacity to sustain long‐term multi‐lineage haematopoiesis. Following up screening and functional validation of NA targets in MPPs and MPs could open up the opportunity searching for regulators conferring long‐term multi‐lineage haematopoiesis capacity on progenitor cells.

In summary, our data show that NA‐overexpressing MPPs can support long‐term multi‐lineage haematopoiesis in primary mice through generating abundant lineage‐committed progenitors, which will benefit the application of MPPs in the absence of LT‐HSCs to improve clinical outcomes in transplantation settings.

## CONFLICT OF INTEREST

The authors declare no conflict of interest.

## AUTHOR CONTRIBUTIONS

YD, KW, QW, TW, PZ, XL, YG, LL and HW performed the experiments and analysed data. JD and JW conceived and supervised the study, wrote the manuscript and approved the final manuscript.

## Supporting information

Fig S1‐S7

Table S1

Table S2

## Data Availability

The data that support the findings of this study are available from the corresponding author upon request. All RNA‐Seq data are in the GEO database with accession code GSE146781.
