# Supplementary material for: NUP98‐HOXA10hd fusion protein sustains multi‐lineage haematopoiesis of lineage‐committed progenitors in transplant setting
Source: Cell Prolif. 2020 Jul 29;53(9):e12885. doi: 10.1111/cpr.12885 (PMC7507399; doi:10.1111/cpr.12885)
Supplement: Supplementary file 1 — Fig S1‐S7 [file CPR-53-e12885-s001.docx]

**Supplementary Figures.**

**
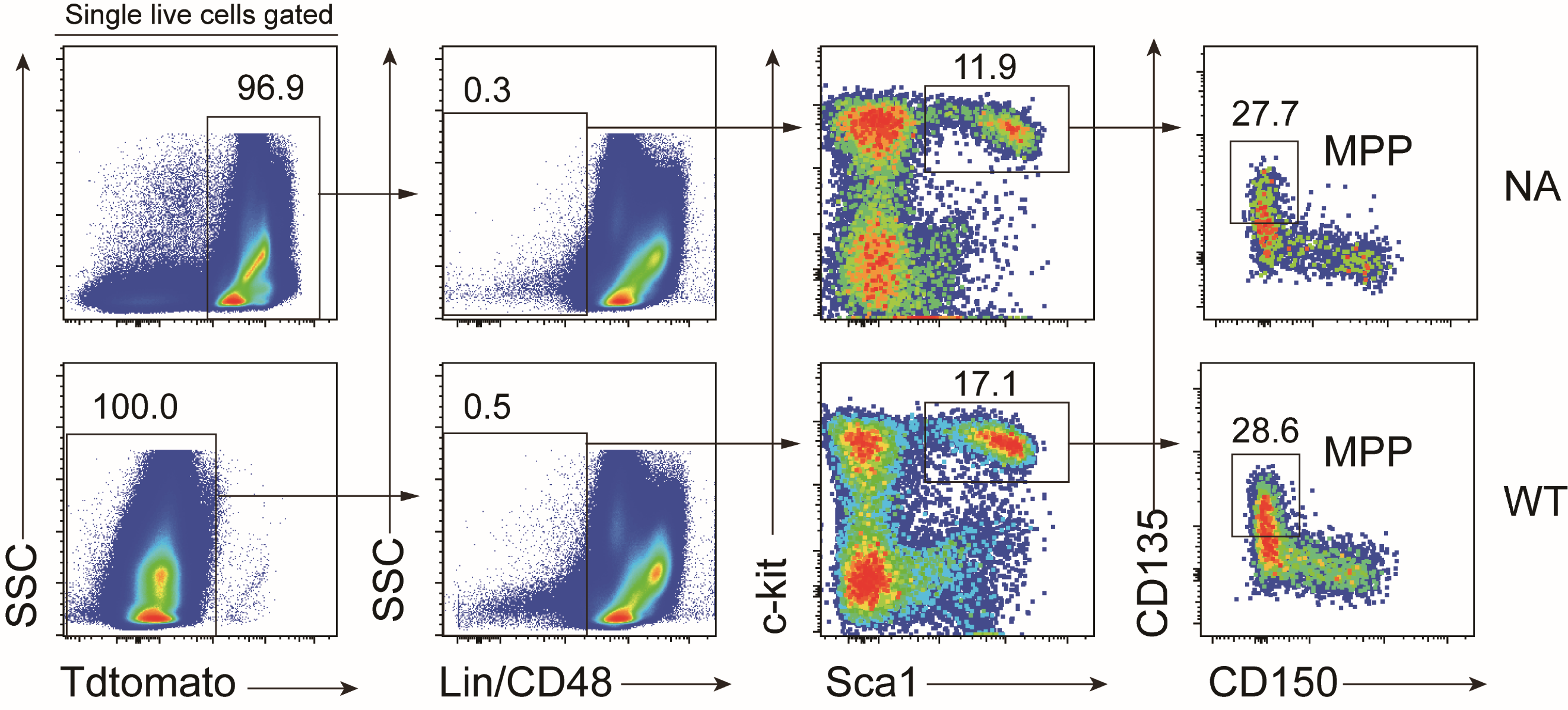
**

**FIGURE S1 Sorting strategy of MPPs from NA and WT mice.**

**
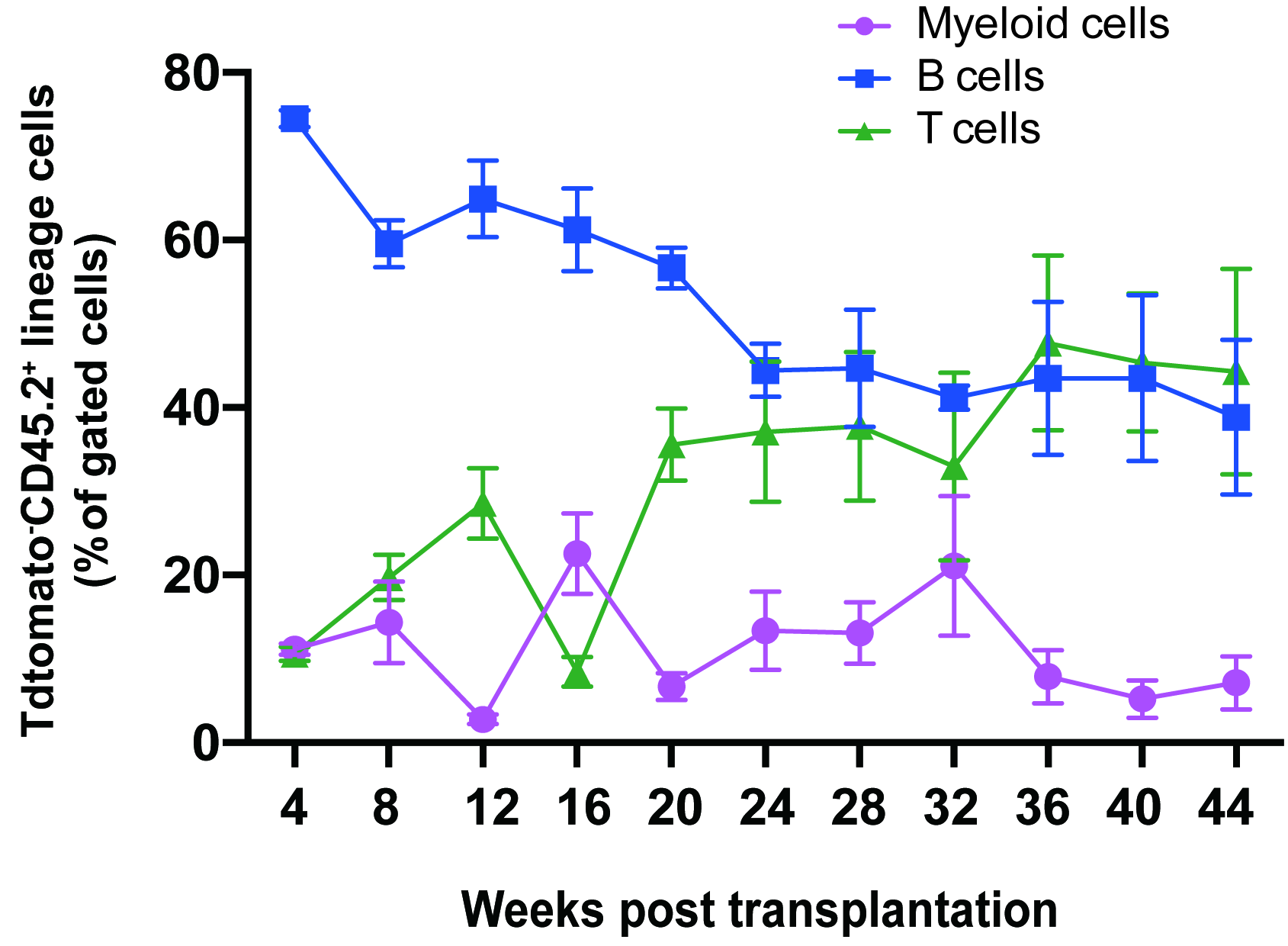
**

**FIGURE S2 Dynamic non-NA-derived multi-lineage reconstitution for NA MPPs recipient mice.** For MPP transplantation assay, 300 sorted MPP cells (Lin^-^CD48^-^ckit^+^Sca1^+^CD135^+^CD150^-^) were mixed with 5🞨10^5^ total BM helper/competitor cells and subsequently injected into the retro-orbital vein of the irradiated recipients. Four mice in each group were bled regularly up to 44 weeks post transplantation. Data are represented as means ± SEM.


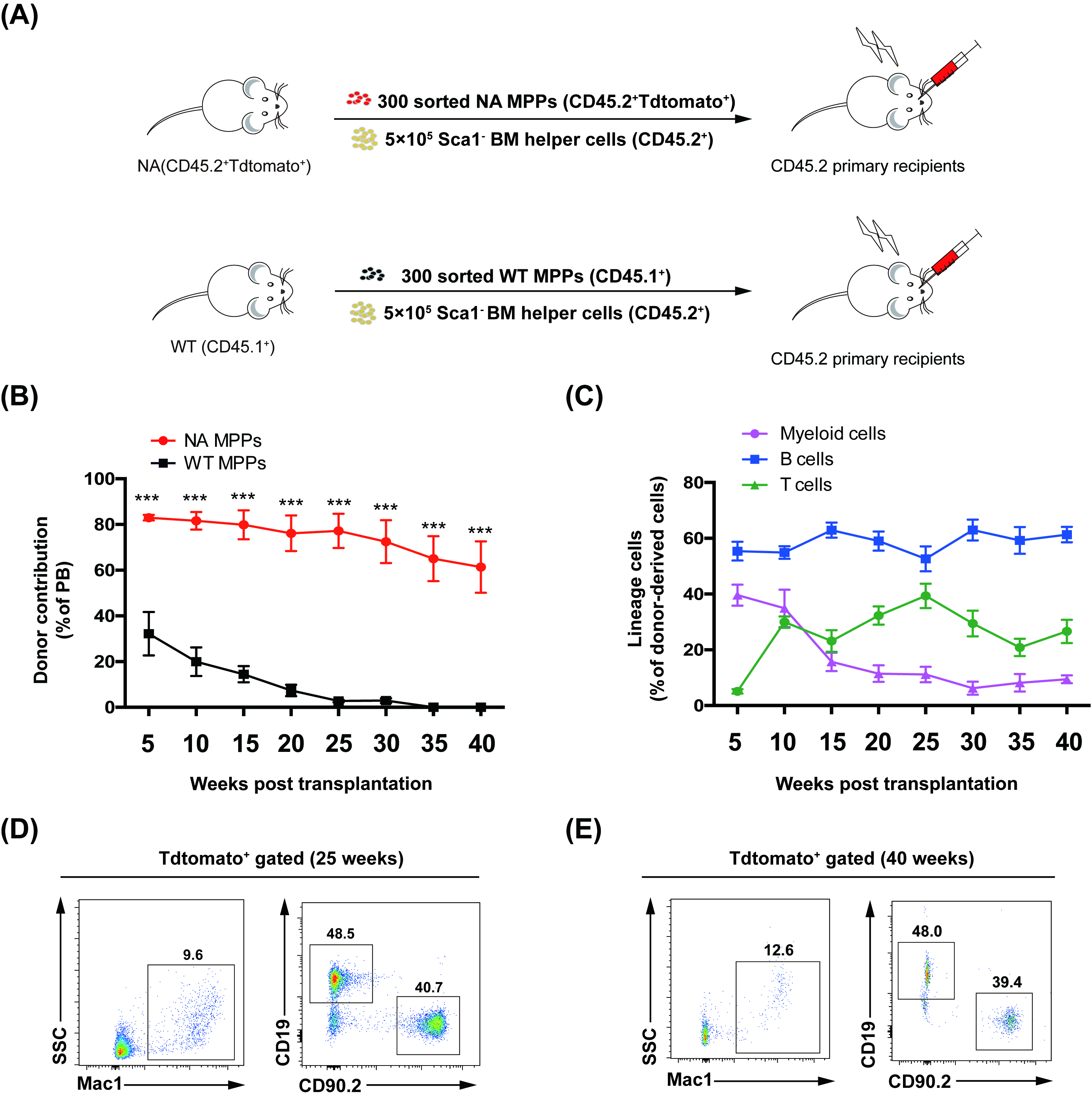


**FIGURE S3 MPPs overexpressing NA support long-term multi-lineage hematopoiesis in primary recipient mice with Sca1^-^ bone marrow helper cells.** (A) Schematic strategy of MPP transplantation. For MPP transplantation assay, 300 sorted MPP cells (Lin^-^CD48^-^c-kit^+^Sca1^+^CD135^+^CD150^-^) were mixed with 5🞨10^5^ Sca1^-^ BM helper cells and subsequently injected into the retro-orbital vein of the irradiated recipients. (B) Dynamic contribution of donor-derived white blood cells (Tdtomato^+^ or CD45.1^+^) in peripheral blood of recipient mice at different time points post transplantation. (C) Dynamic donor-derived multi-lineage reconstitution for NA MPPs group in (B). Four mice in each group were bled regularly up to 40 weeks post transplantation. Lineage analysis of donor-derived white blood cells in PB at 25 weeks (D) and 40 weeks (E) post transplantation. Flow plots from one representative mouse of NA MPPs group are shown. Data are represented as means ± SEM. Unpaired Student’s t-test (two-tailed) was performed. n = 9 mice, *** p<0.001.

**
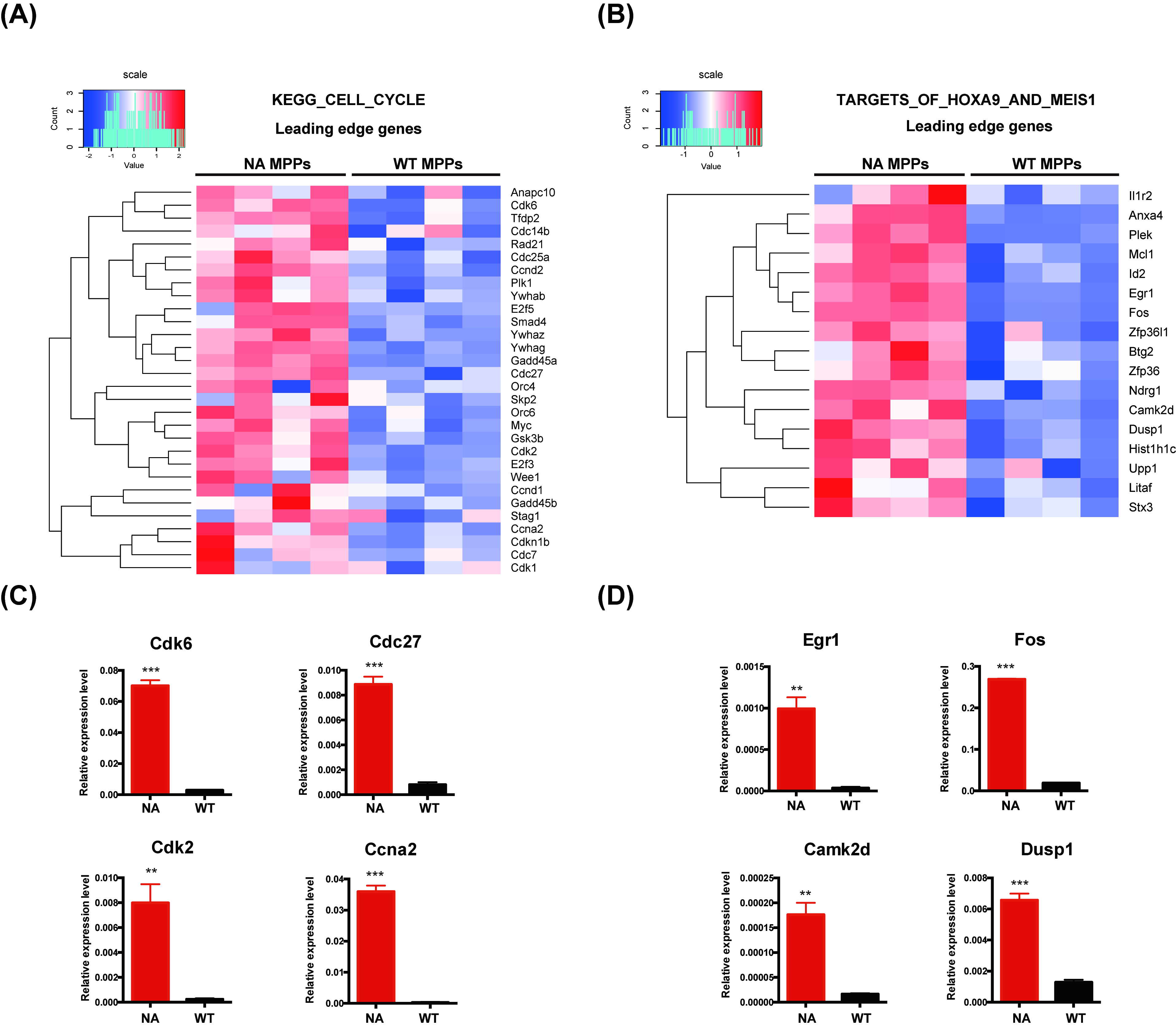
**

**FIGURE S4 Leading edge genes of GSEA pathway of cell cycle and targets of Hoxa9 and Meis1.** (A) Heatmaps represent the expression of leading-edge genes in KEGG_Cell_Cycle pathway. (B) Heatmaps represent the expression of leading-edge genes in targets_of_Hoxa9_and_Meis1 pathway. (C) Real-time PCR validation of selected representative genes from leading-edge gene list of KEGG_Cell_Cycle pathway in NA MPPs and WT MPPs. (D) Real-time PCR validation of selected representative genes from leading-edge gene list of targets_of_Hoxa9_and_Meis1pathway in NA MPPs and WT MPPs. Data from three replicates were plotted and are represented as means ± SEM. Expression relative to the reference gene (Actb) was calculated using the comparative method 2^−ΔCt^. ** p<0.01, *** p<0.001.


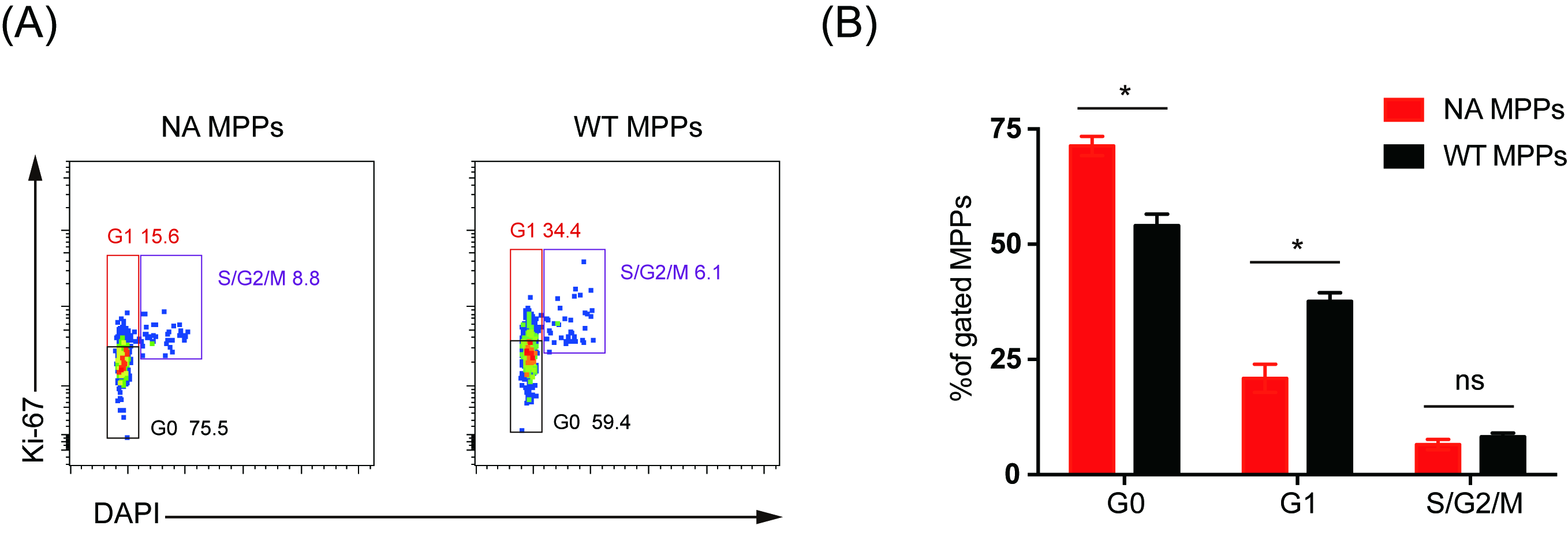


**FIGURE S5 Overexpression of NA alters cell cycle status of MPPs.** (A) Cell cycle analysis of NA MPPs and WT MPPs in bone marrow of NA and age-matched WT mice. Flow plots from one representative mouse of each group are shown. (B) Statistical analysis of cell cycle status of NA MPPs and WT MPPs. n=3 mice. Data are represented as means ± SEM. * p < 0.05. ns, not significant.
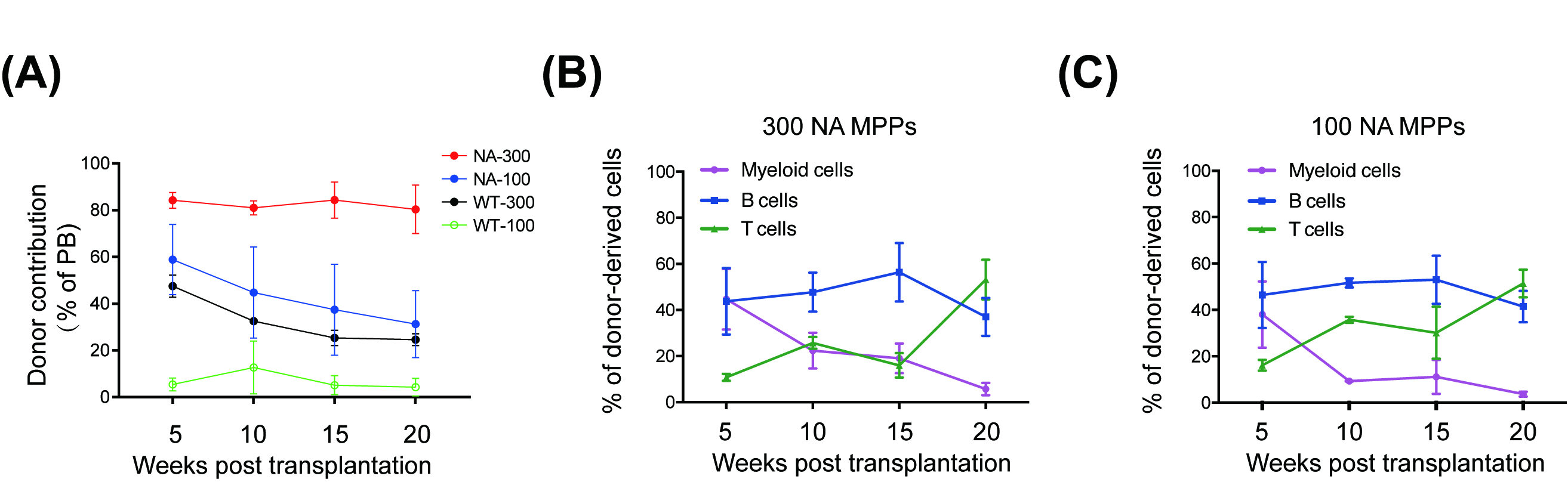


**FIGURE S6 One hundred of NA MPPs failed to maintain steady level of donor contribution when transplanted with Sca1- bone marrow helper cells.** For MPP transplantation assay, 100 or 300 sorted MPP cells (Lin^-^CD48^-^c-kit^+^Sca1^+^CD135^+^CD150^-^) were mixed with 5🞨10^5^ Sca1^-^ BM helper cells and subsequently injected into the retro-orbital vein of the irradiated recipients. Recipient mice were bled regularly up to 20 weeks post transplantation. (A) Dynamic contribution of donor-derived white blood cells (Tdtomato^+^ or CD45.1^+^) in peripheral blood of recipient mice at different time points post transplantation. (B-C) Dynamic donor-derived multi-lineage reconstitution for 300 NA MPPs group (B) and 100 NA MPPs group (C) in (A). Four or three recipient mice were transplanted and followed up respectively for NA MPPs and WT MPPs group. Data are represented as means ± SEM.


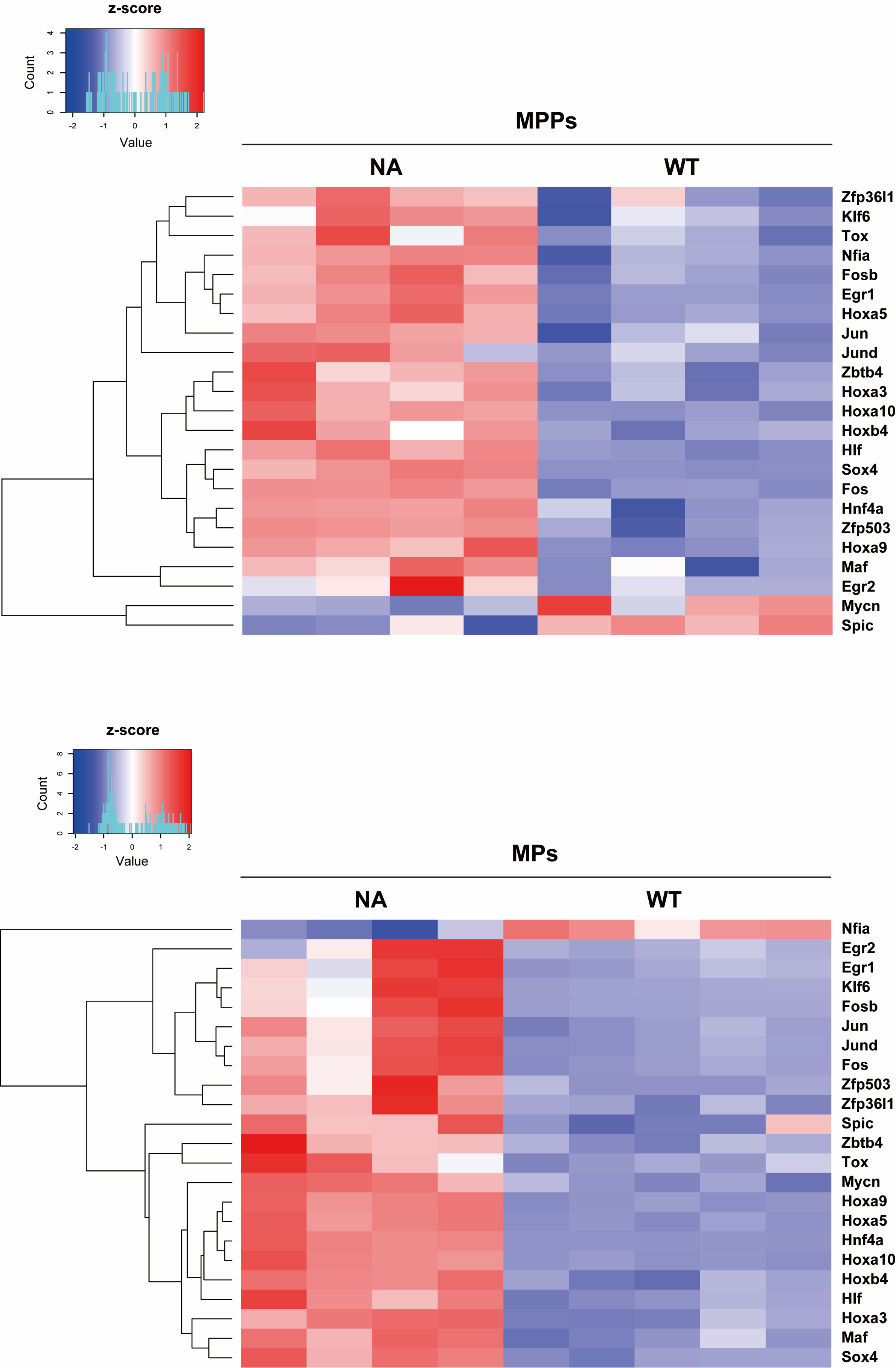


**FIGURE S7 Heatmaps of overlapped transcription factors with more than 1.5-fold change in NA MPPs and MPs over their WT counterparts.**
